# Impact of bacteriocin-producing strains on bacterial community composition in a simplified human intestinal microbiota

**DOI:** 10.3389/fmicb.2023.1290697

**Published:** 2023-12-08

**Authors:** Natalia S. Ríos Colombo, Mariana Perez-Ibarreche, Lorraine A. Draper, Paula M. O’Connor, Des Field, R. Paul Ross, Colin Hill

**Affiliations:** 1 APC Microbiome Ireland, University College Cork, Cork, Ireland; 2 Teagasc Food Research Centre, Moorepark, Fermoy, Co., Cork, Ireland; 3 School of Microbiology, University College Cork, Cork, Ireland

**Keywords:** bacteriocin, pediocin, lantibiotic, lacticin, nisin, microbiome, intestinal microbiota, SIHUMI

## Abstract

Bacteriocins are antimicrobial peptides that have been studied for decades as food bio-preservatives or as alternatives to antibiotics. They also have potential as modulators of the gut microbiome, which has been linked to human health. However, it is difficult to predict *a priori* how bacteriocins will impact complex microbial communities through direct and indirect effects. Here we assess the effect of different bacteriocin-producing strains on a Simplified Human Intestinal Microbiota (SIHUMI) model, using a set of bacteriocin-producing strains (Bac+) and otherwise isogenic non-producers (Bac−). Bacteriocins from different classes and with different activity spectra were selected, including lantibiotics such as lacticin 3147 and nisin A, and pediocin-like bacteriocins such as pediocin PA-1 among other peptides. SIHUMI is a bacterial consortium of seven diverse human gut species that assembles to a predictable final composition in a particular growth medium. Each member can be individually tracked by qPCR. Bac+ and Bac− strains were superimposed on the SIHUMI system, and samples were taken at intervals up to 48 h. The genome copy number of each SIHUMI member was evaluated using specific primers. We establish that the composition of the community changes in response to the presence of either broad- or narrow-spectrum bacteriocin producers and confirm that there are significant off-target effects. These effects were analyzed considering antagonistic inter-species interactions within the SIHUMI community, providing a comprehensive insight into the possible mechanisms by which complex communities can be shaped by bacteriocins.

## 1 Introduction

The gut microbiome is the complex community of microorganisms residing in the human gastrointestinal tract, responsible for numerous functions impacting human health and well-being (Rooks and Garrett, 2016; Tropini et al., 2017). Alterations in intestinal microbial composition are associated with a range of diseases, from acute infections to chronic disorders (Guinane and Cotter, 2013; Gilbert et al., 2018; Heilbronner et al., 2021). This clear link between the microbiome and health has led to an urgent need to develop tools that could be used to predictably shape the gut microbiota.

Bacteriocins are antimicrobial peptides produced by bacteria of many genera that display narrow or broad spectrum of activity (Cotter et al., 2013; Ríos Colombo et al., 2017; Johnson et al., 2018). Many bacteriocin-producing strains are generally regarded as safe (GRAS) or have qualitative presumption of safety (QPS) status and can be considered safe for human consumption. For many years the study of bacteriocins has largely focused on their use as food bio-preservatives or as alternatives to antibiotics (Silva et al., 2018; O’Connor et al., 2020). However, as many members of the human microbiome produce bacteriocins to aid competition in an environment with many competitors for scarce resources, bacteriocins are also considered natural modulators of the human microbiome, and this is one reason why they are gaining interest as potential microbiome-editing tools (Bäuerl et al., 2017; Dicks et al., 2018; O’Connor et al., 2020; Heilbronner et al., 2021).

Bacteriocins are highly diverse in structure, mechanism of action, and spectrum of inhibition. Yet, we do not fully appreciate the potential impact of bacteriocins in specific microbiome contexts. Narrow or broad-spectrum bacteriocins could be expected to exert significant off-target effects when considering interactions among community members competing within the same niche (Guinane et al., 2016; Baquero et al., 2019; Bitschar et al., 2019). The unforeseen consequences of bacteriocin production on the overall structure and function of complex microbial communities remain to be elucidated and rigorous and reproducible evidence is required to assess how bacteriocins could potentially be used as precise microbiome-editing tools. However, the study of the human microbiome is limited both by its complexity and its diversity among individuals.

Different approaches have been used to study the impact of specific bacteriocins on the structure and function of microbial communities, including *in vivo* studies in animal models (Murphy et al., 2013; Bäuerl et al., 2017) and *ex vivo* fermentation of human faecal samples (Guinane et al., 2016). Although these approaches offer different levels of resolution and insight, they come with a number of limitations. The microbiomes of animal models are often too complex and variable to assess specific mechanistic processes, inter-individual heterogeneity in the human faecal microbiota is a significant confounder in most experimental protocols, and data analysis protocols can vary, among many other variables. In this context, *in vitro* defined polymicrobial communities are a useful resource to obtain reproducible data in a controlled experimental setting.

This study set out to assess the effect of different bacteriocin-producing strains on the composition of a SImplified HUman intestinal MIcrobiota (SIHUMI). SIHUMI is a defined bacterial consortium composed of seven culturable and fully sequenced human-derived enteric strains that can be individually tracked in a complex medium using qPCR (Eun et al., 2014; Buttimer et al., 2022). We chose to use interventions consisting of bacteriocin-producing and non-producing strains over the use of pure peptides for two main reasons; (1) bacteriocin-producing organisms could provide an economic and effective means of delivering bacteriocins in the anaerobic environment of the gut, and (2) the use of bacteriocin-producing cultures in functional foods would be subject to less regulatory oversight (Johnson et al., 2018).

We constructed a panel of *Lactococcus lactis* strains that produce bacteriocins (Bac+) from different classes with different inhibitory spectra, including pediocin-like peptides and lantibiotics. The corresponding isogenic non-producer strains (Bac−) were included as negative controls. The Bac+ and Bac− strains were introduced and community composition was evaluated by qPCR to determine the behaviour of the SIHUMI consortium over time. The pediocin-like bacteriocins included pediocin PA-1, pediocin 20336a, hordeiocin, ruminicin, agilicin, and penocin A. These peptides are classified as class IIa bacteriocins, a subgroup of single linear peptides that do not undergo extensive post-translational modifications and have a highly conserved “YGNGV” N-terminal sequence. Pediocin-like bacteriocins are relatively narrow spectrum and display high levels of activity against *Listeria* species. They bind to the mannose phosphotransferase transport system in sensitive strains inducing pore formation leading to cell death (Barraza et al., 2017; Ríos Colombo et al., 2019). The chosen lantibiotics were lacticin 3147 and nisin A. Lantibiotics are a subgroup of class I bacteriocins (post-translationally modified peptides). Unlike pediocin-like bacteriocins, lantibiotics exhibit broad-spectrum inhibition of gram-positive bacteria and they target the lipid II component of the bacterial cell wall inducing both membrane pore formation and inhibition of peptidoglycan biosynthesis (Field et al., 2012; Medeiros-Silva et al., 2018).

In this work, we assessed changes in the SIHUMI assembly after exposure to the different bacteriocin-producing strains. Our results show differential alterations in the composition of the community, impacting both targeted and non-targeted strains. These effects were analyzed considering antagonistic inter-species interactions within SIHUMI, providing a comprehensive insight into the possible mechanisms by which bacteriocin-producing strains might shape human gut microbial communities.

## 2 Materials and methods

### 2.1 SIHUMI strains

SIHUMI is a widely used model with varied composition across several studies like SIHUMIx or SIHUMI-6 (Becker et al., 2011; Krause et al., 2020; Buttimer et al., 2022). In this work we use the SIHUMI consortium described by Eun et al., which consists of *Escherichia coli* LF82, *Enterococcus faecalis* OG1RF, *Lactiplantibacillus plantarum* WCFS1, *Faecalibacterium prausnitzii* A2-165, *Bifidobacterium longum* ATCC 15707, *Phocaeicola vulgatus* DSM1447 and *Ruminococcus gnavus* ATCC 29149 (Eun et al., 2014). These are seven well-characterized and fully sequenced human-derived intestinal bacteria, that form a stable community in wild-type mouse intestine under gnotobiotic conditions. Some of these strains are fastidious anaerobes and can be challenging to grow in the laboratory, especially *F. prausnitzii*, *R. gnavus*, and *P. vulgatus*. We found that LYHBHI medium is both simple to prepare and allows the growth of all seven strains in pure culture. LYHBHI was prepared as described by Sokol et al., 2008: brain–heart infusion medium (Oxoid) supplemented with 0.5% yeast extract (Sigma-Aldrich), 5 mg/L hemin (Sigma–Aldrich), 1 mg/ml cellobiose (Sigma–Aldrich), 1 mg/ml maltose (Sigma–Aldrich), and 0.5 mg/ml cysteine (Sigma–Aldrich; Sokol et al., 2008). All strains were grown in solid and liquid LYHBHI medium at 37°C in strict anaerobic conditions, and were maintained as single-use glycerol stocks at −80°C for long periods. Interactions among the strains were assessed by a cross-streaking method. Overnight liquid cultures of each strain were used to create a vertical streak on LYHBHI agar plates and perpendicular streaks of each of the other strains were simultaneously made, thus obtaining all pairwise combinations. Plates were incubated for 48 h before assessing the results (this assay was performed in triplicate).

### 2.2 Growth analysis of SIHUMI consortium by quantitative real-time PCR

**Figure 1:**
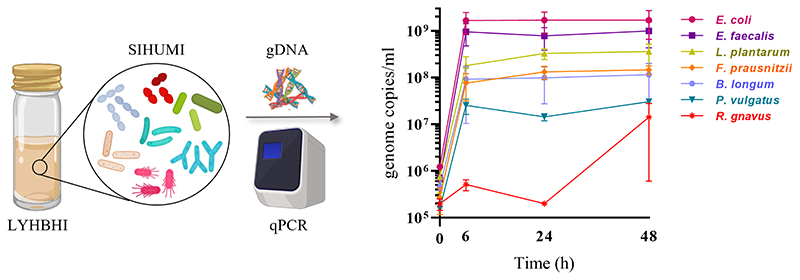
SIHUMI strains were inoculated in LYHBHI and monitored for 48 h using qPCR to quantify the genome copies/ml of each member over time (0, 6, 24 and 48 h). Each time point is represented as a mean with standard deviation of four replicates. Created with BioRender.com.

The growth of each SIHUMI strain was determined by quantitative real-time PCR (qPCR). Each bacterial strain of SIHUMI was grown individually in 5 ml of LYHBHI from a single colony. Each culture was incubated at 37°C in an anaerobic chamber for 24 h and optical density at 600 nm wavelength (OD_600_) was measured by spectrophotometry (Biowave WPA CO8000, Avantor). ODs were standardized to 1 (by dilution with LYHBHI) and 10 μl of each suspension was used to inoculate 10 ml of LYHBHI, constituting the initial SIHUMI consortium. One ml samples were taken at different time points (0, 6, 24, and 48 h post-inoculation). Samples were immediately centrifuged at 7,000 rpm for 2 min to separate the cell pellet from the supernatant, and both were stored at −20°C. Thawed cell pellets were used later for total genomic DNA (gDNA) extraction using the GenElute Bacterial Genomic DNA Kit (Sigma Aldrich, Arklow, Ireland) as described by the manufacturer (Figure 1) with a final elution volume of 200 μl per sample.

Selective species-specific primers for each strain (Lawley et al., 2017; Lengfelder et al., 2019; Guerin et al., 2021; Buttimer et al., 2022) were used for their detection by qPCR within the mixed SIHUMI population (Table 1). Primer specificity was assessed by PCR including a positive and negative control in every run. qPCR was performed in 96-well plates using the QuantStudio™ 5 Real-Time PCR instrument (Applied Biosystems, Thermo Fisher Scientific). Each reaction was carried out in 20 μl volumes containing 1 × SYBR ChamQ Universal SYBR qPCR Master Mix (Generon), 0.4 μM of each primer (forward and reverse), and 1 μl of gDNA. The thermocycling protocol consisted of initial denaturation at 95°C for 2 min, followed by 45 cycles of 95°C for 15 s, 60°C for 15 s and 72°C for 15 s. Standard curves for each bacterial strain were included in every assay plate as well as a negative control where template DNA is replaced with PCR-grade water.

Standard curves were prepared following the Applied Biosystems guidelines (Biosystems, 2013). Briefly, gDNA was extracted from individual strains cultures and concentration (ng/μl) was measured using the Qubit Broad Range assay (Invitrogen/ ThermoFisher Scientific). The mass of each chromosome (one genome copy) was calculated multiplying each genome size (bp) by 1.096 × 10^−12^ (average Molecular Weight of 1 bp in ng). The calculated masses for each genome are listed in Supplementary Table 1. Standard curves for each strain were prepared in a way that the highest concentration is 3 × 10^6^ copies/μl, using the following formula: $x=\frac{3\times 10^{6}\times \text{genome}\;\text{mass}(\text{ng})\times 100}{\text{gDNA}\;\text{concentration}(\text{ng}/\mu \text{l})}$, where *x* is the stock volume of gDNA needed to prepare 100 μl of a 3 × 10^6^ copies/μl solution. Serial dilutions were made to obtain 3 × 10^5^, 3 × 10^4^, 3 × 10^3^, 300, 30, and 3 copies/μl. One μl of each dilution was used as a template in the qPCR reaction to measure the cycle threshold value (Ct). Ct values were plotted as a function of the number of copies/μl to obtain the standard curves for each strain. Genome copy numbers of individual strains in the extracted gDNA from the mixed SIHUMI culture at each time point were calculated as copies/μl based on the measured Ct values. The number of copies/ml was estimated by multiplying the copies/μl by 200 (final elution volume of gDNA extracted from 1 ml of culture sample).

### 2.3 Bacteriocin-producing strains

We chose from our culture collection a set of bacteriocins from different classes and spectrum ranges that could be efficiently produced by the same bacterial host (*Lactococcus lactis* MG1363) to assess the impact of bacteriocin-producing strains in the SIHUMI mix. Some of these strains have been previously described while others were created during this study. Table 2 provides a list of the different bacteriocin-producing strains (Bac+) used in the study, together with their non-producing derivatives (Bac−) lacking the bacteriocin structural genes, and the indicator-sensitive strains.

In previous work, our group developed a set of pNZ44-derived plasmids that functionally express different pediocin-like bacteriocins in *Lactobacillus paracasei* NFBC338 under the control of p44, a strong constitutive promoter (Collins et al., 2018). These plasmids (designated as APC plasmids) contain the structural genes for different pediocin homologues (pediocin PA-1, ruminicin, hordeiocin, agilicin, pediocin 20336a and penocin A) cloned as a fusion with the pediocin PA-1 leader sequence along with *pedD*, a gene encoding the pediocin PA-1 transporter (PedD). PedD is an ABC transporter that recognizes and cleaves the leader sequence while exporting the bacteriocin outside the cell. In this work, these pNZ44-derived plasmids were transformed by electroporation into *L. lactis* MG1363 competent cells (Table 2) following the protocol described by Holo and Nes (1989). Transformants were grown overnight in M17 agar (Oxoid) with 0.5% glucose (GM17) medium containing 5 μg/ml of chloramphenicol (Sigma Aldrich, Arklow, Ireland) for plasmid selection. The same strain was transformed with an empty pNZ44 plasmid for use as a Bac− control (Ped−).

We also selected *L. lactis* strains expressing either of two lantibiotics, lacticin 3147 and nisin A. pMRC01 is a lactococcal plasmid that contains lacticin 3147 genetic determinants and pOM02 is a high-copy-number vector that provides additional genetic determinants for the biosynthetic machinery of lacticin 3147. We used an MG1363 strain harbouring pMRC01 (Ltn+) and the same strain harbouring both pMRC01 and pOM02 (Ltn++), a combination that results in the overproduction of lacticin 3147 (Cotter et al., 2006). A strain with a pMRC01 derivative lacking the two structural genes, *ltnα*/*Itnβ* (Cotter et al., 2003) was used as a Bac− control (Ltn−). *L. lactis* NZ9700 is a nisin-producing strain (Nis+) where the expression of *nisA* (structural gene) is autoregulated by the mature nisin A peptide. *L. lactis* NZ9800 is an NZ9700 derivative carrying a deletion in the structural *nisA* gene that abolishes nisin production (Nis−). Both strains are MG1363 derivatives containing the nisin A biosynthetic gene cluster integrated into the chromosome (de Ruyter et al., 1996). Indicator sensitive strains *L. innocua* DPC3572 and *L. lactis* MG1614 were transformed with pNZ44-E, which provides erythromycin resistance.

Unless otherwise stated, all Bac+, Bac− and indicator strains were routinely grown on GM17 or LYHBHI with the corresponding antibiotic, either in liquid broth or agar plates, at 30°C, under aerobic conditions.

### 2.4 Evaluation of bacteriocin production and activity

Bac+ and Bac− strains were grown overnight in LYHBHI without antibiotics, at 37°C in anaerobiosis, and 1 ml sample of each culture was centrifuged at 7,000 rpm for 2 min to obtain the supernatant. Antimicrobial activity from these supernatants was evaluated by the agar spot diffusion method against a suitable indicator organism (*L. innocua* DPC3572 for pediocin-like bacteriocins and *L. lactis* MG1614 for lantibiotics). Indicator strains harboring the pNZ44-E plasmid enable the addition of erythromycin to media to prevent the growth of remaining bacterial cells in the supernatant. Fifty μl of an overnight culture of the indicator strain was added to LYHBHI medium containing 0.75% agar and 5 μg/ml of erythromycin (Sigma Aldrich, Arklow, Ireland). Ten μl of each supernatant was spotted and the plates were incubated overnight at 30°C in aerobic conditions. Crude supernatants of Bac+ and Bac− strains were also tested against each of the seven SIHUMI members using LYHBHI medium without erythromycin, and plates were incubated overnight at 37°C in anaerobic conditions. The activity of crude supernatants of SIHUMI members was also evaluated against both indicator strains (Supplementary Figure 3).

### 2.5 Detection of bacteriocins by mass spectrometry

Bacteriocin identification was also confirmed by MALDI TOF colony mass spectrometry using a Bruker Autoflex MALDI TOF mass spectrometer (Bruker, Bremen, Germany) in positive-ion reflectron mode (Supplementary Figure 1). Given that the masses of some pediocin-like bacteriocins were not identified by colony mass spectrometry, a two-step purification procedure of cell free supernatants was carried out for ultimate bacteriocin detection. Forty-five ml of supernatants from each sample were applied to an Econo column (Bio-Rad Laboratories, California, USA) containing 5 ml of SP Sepharose Fast Flow (GE Healthcare, Uppsala, Sweden). The column was washed with 20 ml of 20 mM sodium acetate buffer, pH 4.4 containing 0.1 M NaCl, and bacteriocins eluted with 20 ml of 20 mM sodium acetate, pH 4.4, containing 1 M NaCl. The bacteriocin containing eluents were later desalted and concentrated. Eluents were applied to a 3 ml, 200 mg Strata® C18-E SPE column (Phenomenex, Cheshire, UK) pre-equilibrated with methanol and water. The columns were washed with 3 ml of 15% ethanol and then 3 ml of 70% 2-propanol - 0.1% trifluoroacetic acid (IPA). All eluents were assayed against *Listeria innocua* showing no activity was lost in the column flow throughs or washes except for the IPA eluent. An aliquot of each sample was concentrated and assayed for detection of bacteriocins by MALDI TOF mass spectrometry (Supplementary Figure 2). The masses of interest in each sample were detected, suggesting that the bacteriocins were present in the culture supernatants.

### 2.6 Assessment of the effect of bacteriocin-producing strains on SIHUMI

Multiple tubes containing 10 ml of LYHBHI were inoculated with the SIHUMI consortium as described previously. Overnight cultures of the Bac+ and Bac−strains were standardized to a final OD_600_ of 1, and 10 μl of each suspension was added to different SIHUMI-containing tubes at time 0 h. A culture containing just the SIHUMI mix without the Bac+ or Bac− strain was also used as a control. One ml samples were taken at 0, 6, 24, 48 h and processed as described before to obtain supernatants and bacterial gDNA. Quantification of strains’ genome copy numbers at each time point was performed by qPCR using selective species-specific primers as described previously, to assess how the abundance of each bacterial species changed over time upon addition of different bacteriocin-producing *L. lactis*. Additionally, selective primers for *L. lactis* MG1363 were designed in this study using the 16S rRNA gene as a target to track this strain in the consortium (Table 1).

Log fold changes of each SIHUMI strain at 48 h were calculated as the difference between the treatment (addition of Bac+ or Bac− *L. lactis* strain) and the control without the *L. lactis* strain. Statistical analyses were performed on four replicates. GraphPad Prism (v8) was used to determine the normality of data using the D’Agostino-Pearson normality test between treatment groups. A comparison of parametrically distributed data was performed using One-way ANOVA for Ltn−/Ltn+/Ltn++ and Ped-/PA1+/20336a+/Hord+/Rum+/Agil+/PenA+ data sets. The mean of each Bac+ treatment was compared to their corresponding Bac− control using the Dunnett test. A two-tailed unpaired *t*-test was used to compare the Nis–/Nis+ data set. Statistical significance was defined as having a *p* value of 0.05 for the comparisons indicated.

Relative bacterial abundances at 48 h from mean genome copies for each bacterium were calculated among all tested conditions using the following formula: [(genome copies for a specific SIHUMI bacterium/total number of genome copies in the mixture) × 100]. The cumulative number of genomes from each species in the mix were considered as the total number of genome copies.

## 3 Results

### 3.1 SIHUMI assembly

Establishing reproducible conditions for the cultivation and individual tracking of microbiome-derived bacteria is a prerequisite for the development of an accurate model. LYHBHI medium was found to be the best option for proper growth of all seven SIHUMI strains, including the fastidious ones. When grown as a consortium, each member strain genome copies in SIHUMI reached reproducible levels at time points up to 48 h (Figure 1). The highest number was achieved by *E. coli* followed by *E. faecalis, L. plantarum, F. prausnitzii, B. longum, P. vulgatus* and finally *R. gnavus*.

**Figure 2:**
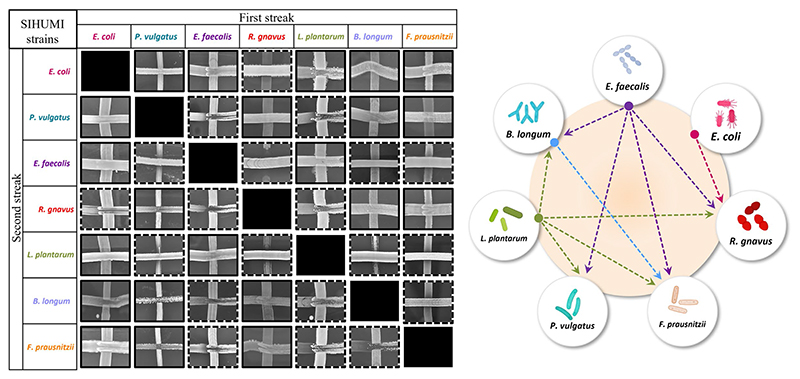
Left: Cross-streaking assay to assess antagonistic interactions among SIHUMI members. A first streak of each SIHUMI strain was vertically applied across an LYHBHI agar plate. On the side, a second streak of each strain was then perpendicularly applied. Antagonism between two strains was considered positive when growth inhibition of one of them was observed in both the first and the second streaks (highlighted in dashed lines). Right: Antagonism network diagram of SIHUMI consortium in LYHBHI. Arrows represent the antagonism exerted by *E. faecalis* (purple), *E. coli* (pink), *L. plantarum* (green) and *B. longum* (blue). The origin of the arrows indicates the antagonizing strain while the arrowheads point to the antagonized strain.

Community composition results from a delicate balance of microbial interactions, the growth rates of the individual strains, and the carrying capacity of the system (Faust and Raes, 2012). Inter-species interactions have been described to be major drivers (Davis et al., 2022), although for complex communities such as human microbiomes the nature of these interactions is typically unknown. However, in a defined community, a simple cross-streak method can be used to qualitatively assess antagonism between microorganisms. Thus, all seven SIHUMI strains were cross-streaked against each other to identify paired inhibitory interactions and draft an antagonism network (Figure 2).

This antagonism network is a useful tool to understand the final assembly of SIHUMI. It shows that *E. faecalis* and *L. plantarum* are the principal inhibitors (each inhibiting four other members of the consortium) while *R. gnavus* and *F. prausnitzii* are highly inhibited by other community members. The underlying mechanisms by which some strains behave as strong inhibitors and others are inhibited are beyond the scope of this study. However, these antagonistic interactions align with the final community composition in that *E. coli*, *E. faecalis* and *L. plantarum* are the most abundant strains in the mix while *R. gnavus* and *P. vulgatus* (which are strongly antagonized by other members) are less abundant after 48 h (Figure 1). It is worth remarking that *R. gnavus* genome copies increase at 48 h relative to 24 h. This might be the result of the concomitant death or metabolic inactivation of its inhibitors (*E. coli*, *E. faecalis* and *L. plantarum*) as they could be reaching a late stationary phase allowing *R. gnavus* to grow only after 24 h.

### 3.2 Antimicrobial activity of Bac+ strains supernatants

**Figure 3:**
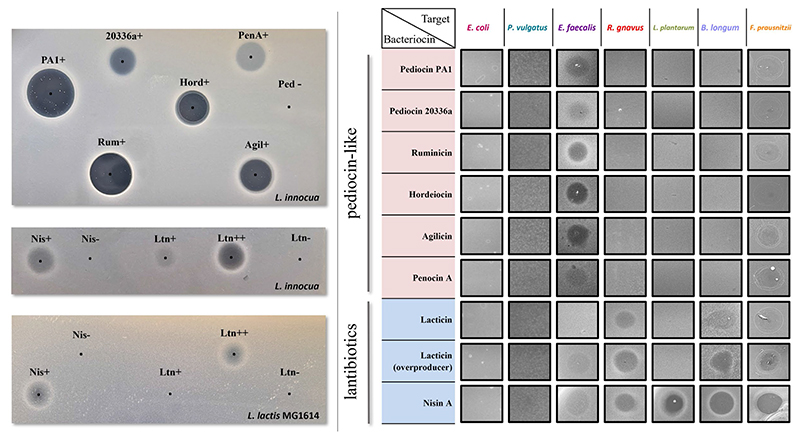
Left: Antimicrobial activity of culture supernatants from Bac+ and Bac− strains by agar spot diffusion method against the indicator organism *L. innocua* DPC3572 and *L. lactis* MG1614. The black dots indicate the centre of each supernatant spot. Right: antimicrobial activity of culture supernatants from Bac+ strains against each of the seven SIHUMI members. None of the Bac− supernatants displayed inhibition halos (data not shown).

All selected bacteriocins are effectively expressed by the producing *L. lactis* host as shown by the inhibition halos displayed by the crude supernatants of the Bac+ strains against sensitive indicators (Figure 3). MALDI-TOF mass spectrometry was also used to confirm bacteriocin production (Supplementary Figures 1, 2).

As with most pediocin-like bacteriocins, the selected pediocin homologues display anti-listerial activity, although the size and clarity of the inhibition halos vary between the different producers. Pediocin PA-1 and pediocin 20336a share 90% identity, despite different activity profiles observed from the crude supernatant. Hordeiocin, ruminicin, and agilicin share between 60 and 76% amino acid identity with pediocin PA-1 while penocin A lacks a high degree of similarity (Collins et al., 2018). Regardless of the percentage identity, increased or decreased zone sizes among these peptides could result from varying production levels, or different physicochemical properties such as enhanced solubility and diffusion, or a higher specific activity (Field et al., 2008). Nonetheless, all crude supernatants displayed bioactivity, therefore, the producers were subsequently tested against the SIHUMI consortium.

Nisin A and lacticin 3147 target both indicator strains *L. innocua* DPC3572 and *L. lactis* MG1614, although the antimicrobial activity of the supernatants is less pronounced than that observed for pediocin-like bacteriocins. The activity of the lacticin 3147-overproducer strain (Ltn++) harbouring pMRC01 and pOM02 is higher than that of the Ltn+ strain containing only pMRC01, as described previously (Cotter et al., 2006).

None of the supernatants of the standard SIHUMI members display activity against the indicator strains except for that of *E. faecalis*, which displays activity only against *L. innocua* (Supplementary Figure 3). As this activity could mask the weak halo produced by the lantibiotic-producing strains in SIHUMI, *L. lactis* MG1614 was selected as the indicator strain to evaluate nisin and lacticin production in the consortium while *L. innocua* was used to evaluate the pediocin-like bacteriocins.

The ability of these bacteriocins to target each member of the consortium was assessed by spot assay of the supernatants against a lawn inoculated by individual SIHUMI strains. After incubation in anaerobic conditions at 37°C, the supernatants only formed inhibition zones on the target hosts (Figure 3). As expected, all the bacteriocins are inactive against *E. coli* and *P. vulgatus* since these peptides do not normally target Gram-negative bacteria. In contrast, all show more or less activity against *E. faecalis*. The lantibiotics target virtually all the gram-positive strains within SIHUMI, except for lacticin 3147 where no inhibition halo is seen against *L. plantarum*. This strain encodes for a putative ABC transporter homologous to LtnFE (lacticin 3147 immunity system) that may provide protection against lacticin (Draper et al., 2009). On the contrary, the pediocin-like bacteriocins are very narrow-spectrum, displaying inhibition halos only against *E. faecalis*.

### 3.3 Impact of bacteriocin-producing *Lactococcus lactis* on SIHUMI

**Figure 4:**
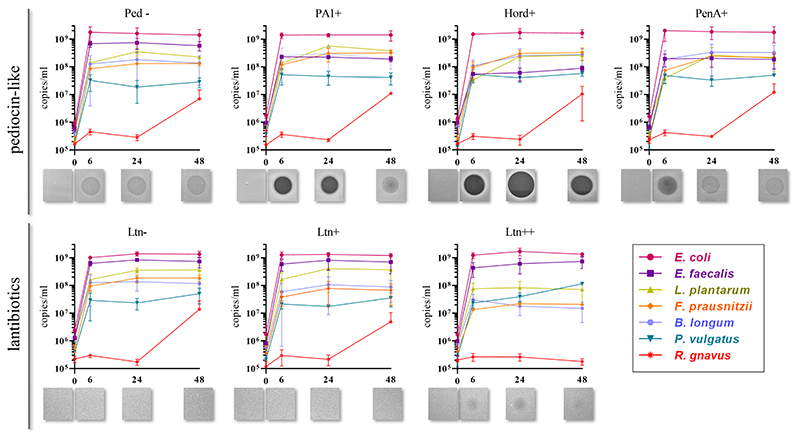
Genome copies/ml over time (0, 6, 24 and 48 h) of members of SIHUMI consortium in LYHBHI after inoculation with Bac+ and Bac− *L. lactis* strains at time 0. Each time point is represented as a mean with standard deviation of 4 replicates. Displayed below each graph are the spot assays of culture supernatants at each time point against *L. innocua* (for pediocin-like bacteriocins) and *L. lactis* MG1614 (for lantibiotics). Ped- (non-producing control), PA1+ (pediocin PA-1), Hord+ (hordeiocin), PenA+ (penocin A). Ltn− (non-producing control), Ltn+ (lacticin 3147), Ltn++ (lacticin 3147 overproducer).

**Figure 5:**
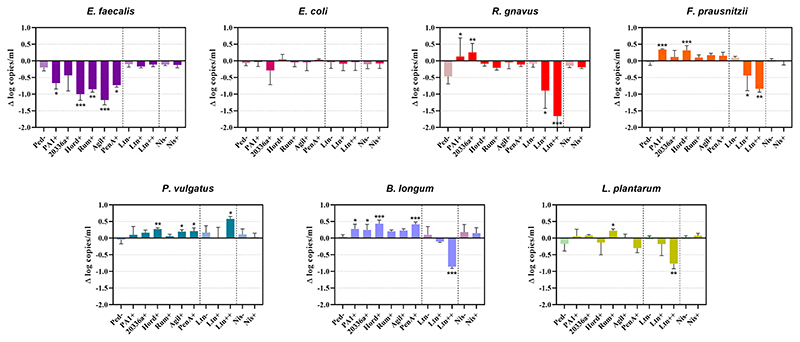
Difference (Δ) in the log of genome copy number of SIHUMI members at 48 h after the addition of Bac+ or Bac− *L. lactis* strain to the consortium. The log change is relative to that of the SIHUMI without *L. lactis* and is represented as the mean of Δ log copies/ml ± standard deviation. Statistical significance was analyzed by comparing each Bac+ treatment to their corresponding Bac− control and was recorded as follows: *** (*p* < 0.001), ** (*p* < 0.01), * (*p* < 0.05), no asterisk means no significant difference (*p* > 0.05). The data presented are the averages of 4 replicates. Ped- (non-producing control), PA1+ (pediocin PA-1), 20336a+ (pediocin 20336a), Hord+ (hordeiocin), Rum+ (ruminicin), Agil+ (agilicin), PenA+ (penocin A). Ltn− (non-producing control), Ltn+ (lacticin 3147), Ltn++ (lacticin 3147 overproducer), Nis− (non-producing control), Nis+ (nisin A).

**Figure 6:**
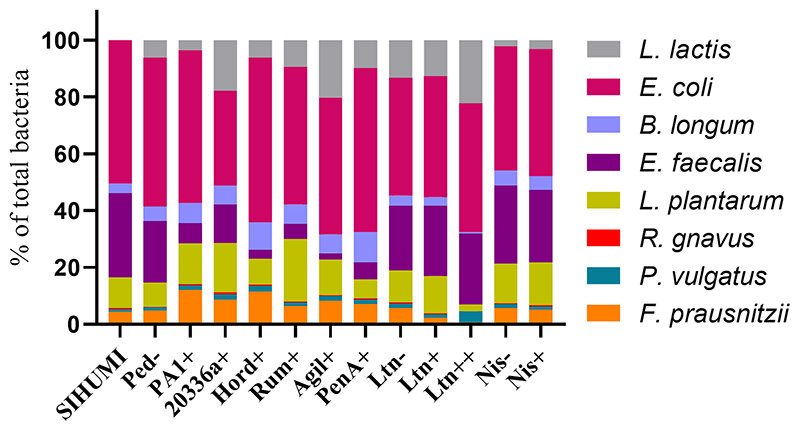
Relative abundance of bacterial members in the SIHUMI consortium treated with Bac+ or Bac− *L. lactis* strains after 48 h. The means of genome copies of each species are represented as a percentage of the total population of the community (cumulative abundances of all strains genome copies).

Quantification of genome copies/ml of SIHUMI members in the consortium was performed by qPCR after addition of either the Bac+ or Bac− strains at time 0 h. Figure 4 shows the genome copy number of each SIHUMI strain in the consortium together with the antimicrobial activity of the supernatant against the corresponding indicator strain at different time points after addition of some of the Bac+ and Bac− *L. lactis* strains. Results for all the tested *L. lactis* strains are shown in Supplementary Figures 4, 5. The log fold change of each strain was calculated 48 h after the different Bac+ and Bac− *L. lactis* were added to the consortium (Figure 5). We also compared the relative abundance of each bacterium in the consortium after 48 h (Figure 6).

Pediocin-like bacteriocins were successfully produced and released in this setting, as shown by the inhibitory zones against *L. innocua* in the consortium supernatants. When *L. lactis* Ped- is added, the consortium supernatants display hazy zones probably due to the inhibitory activity of *E. faecalis* against *L. innocua* (Supplementary Figure 3). However, consortium supernatants containing pediocin-like peptides expressed by *L. lactis* display antimicrobial activity that is consistent with the anti-*Listeria* activities previously reported for these peptides (Collins et al., 2018). At time 6 h, pediocin PA-1 produces a clear halo that remains at 24 h and fades by 48 h. The same pattern is observed for all the pediocin-like peptides. Hordeiocin, ruminicin and agilicin exhibit superior antimicrobial activity profiles compared to pediocin PA-1 while penocin A and pediocin 20336a appear to be reduced to a certain degree (Figure 4; Supplementary Figure 5).

There is a consistent reduction in the levels of *E. faecalis*, the only strain targeted by pediocin homologues, which aligns with the antimicrobial activity observed in the consortium supernatants at different time points (Figure 4). *E. faecalis* numbers clearly decrease when exposed to the different pediocins, and the highest log decrease is caused by hordeiocin, ruminicin and agilicin (Figures 5, 6). Interestingly, over time the levels of other non-targeted members are also affected: *F. prausnitzii*, *P. vulgatus*, *B. longum*, and *R. gnavus* show a simultaneous increase in the presence of all the pediocins (Figure 5). These results are consistent with the antagonism network (Figure 2) that reveals that *E. faecalis* inhibits these four members of the consortium. Even though this increase is only significant for some of the bacteriocin producers, there is a clear trend suggesting that the impact of bacteriocins goes beyond the targeted knockdown of sensitive species within a bacterial consortium.

Lacticin 3147 is not detectable in the supernatants when produced by *L. lactis* Ltn+, although a significant decrease in two of the targeted SIHUMI species is observed (*R. gnavus* and *F. prausnitzii*). When overproduced by *L. lactis* Ltn++, the lacticin 3147 present in the supernatants displays small inhibition halos against the *L. lactis* MG1614 and produces drastic changes in the SIHUMI assembly (Figure 4). The low or null antimicrobial activity of lacticin 3147 in the consortium supernatants (in contrast to that observed for pediocin homologues) might be due to a substantial binding of the peptide to sensitive SIHUMI strains in accordance with its broad-spectrum nature. It is likely that a significant fraction of the bacteriocin remains in the cell pellet after centrifugation decreasing the presence of the peptide in the supernatant. *R. gnavus*, *F. prausnitzii* and *B. longum* numbers dramatically decrease as a result of the lacticin 3147 derived from the overproducer (Figure 5), in agreement with the spot assays (Figure 3). Although some antimicrobial activity against *E. faecalis* by lacticin 3147 is observed in the spot assays, the lantibiotic had no significant impact on this strain within the consortium at 48 h. Conversely, *L. plantarum* is highly inhibited in the consortium by *L. lactis* Ltn++, despite no observable activity when assessed against this strain on agar plates. This suggests that results from agar-based growth inhibition assays do not always correlate with activity against bacteriocin susceptible microbes in a polymicrobial community. Finally, *P. vulgatus* undergoes a significant increase in numbers, and this off-target effect can be explained by the antagonism network since *L. plantarum* inhibits *P. vulgatus*. Thus, the direct reduction of *L. plantarum* results in an indirect benefit for *P. vulgatus* in the consortium.

When the Nis+ strain is added to SIHUMI, nisin A is not detected in the supernatants through the spot assay, and no changes are observed in SIHUMI growth curves compared to the Nis− control (Supplementary Figures 4, 5), despite nisin being considerably active against all the Gram-positive strains of the consortium (Figure 3). This could be explained by the fact that nisin auto-regulates its own expression via the *nisRK* two-component histidine kinase response regulator system. In the nisin-producing strain *L. lactis* NZ9700 (Nis+), the *nis*A promoter tightly regulates transcription based on extracellular nisin levels. Nisin production increases linearly with nisin concentration in the medium, leading to a considerable surge in production after mid-exponential growth. While induction requires low nisin concentrations (below inhibitory levels), in the absence of nisin, the operon remains inactive, resulting in no production (de Ruyter et al., 1996). It might be the case that any residual nisin A resulting from the inoculation medium is attaching to the sensitive SIHUMI members in the consortium, reducing the available extracellular nisin below the levels required for induction. As a result, the genetic switch that controls nisin production remains off. Consequently, no detectable activity is observed in the supernatants or any noticeable changes in the growth patterns of SIHUMI are perceived.

### 3.4 Effect of bacteriocin production on *Lactococcus lactis* growth within SIHUMI

**Figure 7:**
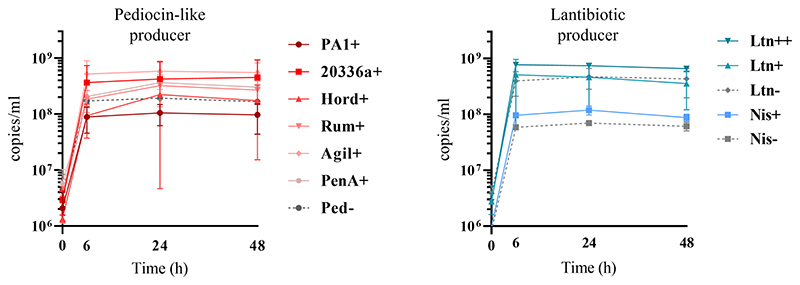
Genome copies/ml over time (0, 6, 24 and 48 h) of Bac+ and Bac− *L. lactis* strains growing within the SIHUMI consortium in LYHBHI. Each time point is represented as a mean with a standard deviation of 4 replicates. Pediocin producer strains: Ped- (non-producing control), PA1+ (pediocin PA-1), 20336a+ (pediocin 20336a), Hord+ (hordeiocin), Rum+ (ruminicin), Agil+ (agilicin), PenA+ (penocin A). Lantibiotic producer strains: Ltn− (non-producing control), Ltn+ (lacticin 3147), Ltn++ (lacticin 3147 overproducer), Nis− (non-producing control), Nis+ (nisin A).

Bacteriocin production also impacts on *L. lactis* behavior in the consortium. The production of bacteriocins has been shown to be one means by which bacterial strains can gain a competitive advantage in complex communities with constant competition for nutrients and space (Kommineni et al., 2015). This might explain why the production of most of the pediocin homologues boosts the numbers of *L. lactis* relative to non-producing controls (Figures 6, 7). However, this is not consistent for all the peptides, as pediocin PA-1 and hordeiocin do not lead to an increase in *L. lactis* levels. On the other hand, lacticin 3147 seems to slightly increase the *L. lactis* number only when it is overproduced, compared to the Ltn− control. A similar behavior is observed for the Nis+ strain although at 48 h the genome copies/ml are no longer significantly different from the Nis− strain.

## 4 Discussion

Precise and predictable manipulation of the human microbiota remains extremely challenging. As of now, the major levers for changing the microbiome composition are “blunt” forces such as dietary changes or antibiotic administration (Relman, 2012). While pre, pro and postbiotics have a well-documented ability to promote the growth of beneficial microorganisms and contribute to the production of advantageous metabolites (Shah et al., 2020; Koc et al., 2022), bacteriocins represent a potential strategy for fine-tuned microbiome modulation through their antimicrobial action. However, the inhibitory spectrum of bacteriocins is usually assessed individually against a limited set of strains and this does not necessarily reflect their likely impact on complex and dynamic communities. In addition, it is not clear how bacteriocin production in the intestine may alter the overall structure and function of the microbiome.

The gut microbiome is an ecosystem where species compete for limited resources such as nutrients and space, and their populations grow and decline in response to the presence of other microbes. Stimulatory, antagonistic, or neutral inter-species interactions are a major determinant of community assembly (Davis et al., 2022). However, in complex communities like microbiomes, determining the varieties and strength of interactions among species is typically the bottleneck that limits our understanding, mainly because the human gut usually contains over 100 species (Qin et al., 2010). This is further complicated by the inherent difficulties associated with *in vivo* and *ex vivo* models. Conversely, a synthetic *in vitro* consortium like SIHUMI allows us to evaluate inter-species interaction given the relative simplicity of the system. We used the cross-streak method to characterize the interactions between the seven SIHUMI members. This method does not provide quantitative data but can rapidly give qualitative information. The resulting SIHUMI antagonism network aligns with the final assembly of the consortium and explains to some extent the changes observed in response to specific bacteriocins. However, further elucidation of other types of inter-species interactions within SIHUMI (mutualistic or neutral), as well as a quantitative evaluation of these interactions are still necessary to gain a deep insight into how bacteriocin production can be integrated with polymicrobial communities. Although there are no consensus or standardized methods to measure these interactions, some recently reported approaches include the use of two-species cultures monitored overtime by OD measurements and multiplexed 16S rRNA gene sequencing to calculate the relative abundance of each species (Venturelli et al., 2018); single-species cultures in cell-free spent media of other species, to quantify how changes in the chemical environment by each strain affects its cohabitants (Dedrick et al., 2023); and dropout communities (top-down approach) by removing one species at a time and compare community behavior in the presence and absence of each member to quantitively elucidate the influence of each organism (Weiss et al., 2023).

In this work, we constructed a panel of *L. lactis* strains that produce broad- and narrow-spectrum bacteriocins targeting different members of the SIHUMI community and demonstrated that these bacteriocins could be effectively produced at 37°C in LYHBHI medium under anaerobiosis, the same growth conditions used to grow the SIHUMI strains.

The selected bacteriocins include pediocin-like bacteriocins and lantibiotics, among which are pediocin PA-1 and nisin A, the prototype peptides of each group, respectively. These peptides have been intensively studied and have a long record of successful and safe use as commercial additives in the food industry for the inhibition of spoilage and pathogenic bacteria (Taylor, 2014; Navarro et al., 2019). We also included a set of pediocin homologues displaying higher or lower potency relative to that of pediocin PA-1 against *Listeria* (Collins et al., 2018) and a two-peptide lantibiotic, lacticin 3147 that displays higher potency relative to that of nisin against multiple pathogens (Iancu et al., 2012).

In general terms, bacteriocin production reduces the abundance of targeted members within the SIHUMI consortium. Nonetheless, there are important off-target effects beyond the bacteriocin-targeted knockdown of sensitive strains. Here we present a comprehensive assessment of bacteriocin production in the context of a consortium of competing bacteria, analyzing the effects on both targeted and non-targeted members:

1. *E. coli* is the dominant member within the community under our conditions. This strain constitutes nearly 50% of the total population after 48 h (Figure 6) and remains consistently stable in the community across the different treatments with the Bac+ strains, showing no substantial changes as compared to the Bac− controls (Figure 5). This can be attributed to the lack of direct inhibitory effects exerted by the tested bacteriocins on *E. coli* (Figure 3). Additionally, there is no observable indirect effect, as its growth seems to be unaffected by the variation in the population of other members of the community.
2. *E. faecalis* is the second most prevalent bacterium after 48 h (Figure 1) and is the strain most affected by the pediocin homologues. *E. faecalis* is a potent antagonistic member within the community (Figure 2), so the direct inhibition of this strain causes indirect increases in multiple other members.
3. *L. plantarum* is another potent inhibitor that is minimally impacted by the pediocin-like bacteriocins. Contrastingly, *L. plantarum* is substantially reduced by the lacticin 3147 overproducer within the consortium (Figure 5). Curiously, this lantibiotic fails to display antimicrobial activity when individually tested against *L. plantarum* (Figure 3), possibly due to the presence of a lacticin 3147 immunity protein homologue (LtnFE). However, this homologue from *L. plantarum* has shown low efficiency for immune mimicry against lacticin 3147 compared to similar homologues from other species (Draper et al., 2009). Moreover, immunity proteins have limitations in providing protection, especially when bacteria are exposed to high levels of the bacteriocin (Ríos Colombo et al., 2017). This highlights how the inhibitory potential of bacteriocins might be underestimated by conventional agar-based screening against single strains.
4. *P. vulgatus*, akin to *E. coli*, is a gram-negative species and thus remains largely unaffected by the tested bacteriocins (Figure 3). However, unlike *E. coli*, *P. vulgatus* benefits from the presence of pediocin(s) and lacticin 3147 producers (Figure 5). These effects arise due to the strong antagonism exerted by *E. faecalis* and *L. plantarum* upon *P. vulgatus*. Although this strain has been linked to opportunistic infections in inflammatory bowel diseases (Eun et al., 2014), it is commonly found in the healthy gut microbiome and its role as either beneficial or harmful is still unclear and requires further investigation.
5. The growth of *B. longum* in the consortium is negatively impacted by lacticin 3147, although it is enhanced by the pediocins (Figure 5), probably due to an indirect consequence of inhibition of *E. faecalis*. This is an interesting outcome worthy of further exploration, given that bifidobacteria are among the predominant bacteria in a healthy human gut and play a significant role in maintaining gut health (O’Callaghan and van Sinderen, 2016).
6. *F. prausnitzi* is highly inhibited by *E. faecalis*, *B. longum* and *L. plantarum* (Figure 2). Though pediocins inhibit *E. faecalis*, boost *B. longum* and minimally impact *L. plantarum*, the resulting balance of interactions leads to an increase in *F. praustnizii* numbers in the presence of these peptides (Figure 5). In contrast, the direct impact of lacticin 3147, when overexpressed, leads to a substantial reduction of *F. prausnitzii* in the consortium by nearly one log unit.
7. Finally, *R. gnavus* is highly inhibited by *E. faecalis* and *L. plantarum*, which might explain the low levels of this strain in the consortium (Figure 1). By direct inhibition of *E. faecalis*, pediocin-producing strains indirectly allow *R. gnavus* to reach higher levels within the community (Figure 5).

These results suggest that SIHUMI behaves in such a way that specific strain-directed knockdowns by different bacteriocins generate a “domino effect” that leads to broader consequences in other members as a result of the inter-species interaction network. While we acknowledge that other types of interactions (such as mutualism or neutralism) might be taking place within the consortium, the pair-wise antagonisms identified in this study are entirely consistent with the perturbations resulting from the introduction of the bacteriocin-producing strains.

Similar off-target effects were reported by Buttimer et al. (2022) when analyzing the effect of phages in a SIHUMI consortium using continuous fermentation systems. In that study, a phage cocktail targeting *E. coli* and *E. faecalis* was shown to significantly alter the abundance of non-target species. Phage predation reduced *E. coli* and *E. faecalis*, causing a concomitant increase in other community members like *L. plantarum* and *F. prausnitzii*. While the experimental conditions for growing the SIHUMI consortium were different in that study, our results reinforce these previous conclusions.

We failed to observe inhibition of SIHUMI members specifically targeted by nisin A when using the Nis+ *L. lactis* strain. This was an unexpected result given the abundance of studies highlighting the capacity of nisin to influence the gut microbiota (for a comprehensive review see Field et al., 2023). Indeed, a recent publication demonstrates that orally-ingested nisin remains intact through porcine gastrointestinal transit, impacting both the composition and functionality of the microbiota (O’Reilly et al., 2023). Moreover, nisin caused a decrease in gram-positive bacteria and a corresponding relative increase in gram-negative bacteria. However, in that report, nisin was applied as a pure peptide at 50 μM while nisin-producing strains produce significantly less (unpublished results). Furthermore, the auto-regulated nisin biosynthesis by the *L. lactis* NZ9700 strain used here, appears to be switched off in the consortium, possibly due to binding of extracellular nisin to the multiple sensitive SIHUMI members. While the nisin-inducible expression system is advantageous for continuous overexpression of nisin and other proteins in industrial settings (de Ruyter et al., 1996), our results suggest that this system might represent a limitation when considering nisin-producing strains for microbiome-targeted intervention. Other expression systems like the constitutive p44 promoter might be a better option for bacteriocin expression, as shown for the pediocin-like peptides.

It is worth noticing that our data were generated by qPCR analysis using species-specific primers. In contrast to 16S rRNA gene sequencing, qPCR provides a more accurate estimation of the number of individual genomes in the sample. This is because the number of 16S gene copies varies between bacterial species, impacting the relative abundance estimation in 16S rRNA gene sequencing-based microbiome analysis. Specialized bioinformatics tools and software can be used to correct for gene copy number variation, but high accuracy levels are usually computationally intensive when dealing with extremely complex samples like fecal microbiomes, leading to incorrectly identifying certain species as dominant or overlooking certain others due to underestimated abundances. Within defined communities, qPCR allows a more refined detection of genome copy numbers by using standard curves, providing a quantitative assessment of the gene copy number variation.

Taken together, our results demonstrate that a simplified system like SIHUMI facilitates the behavioral monitoring of individual strains within the consortium with a high level of reproducibility, mitigating inter-individual variation that can occur in studies using human faecal microbiota. Furthermore, the impact of bacteriocins on SIHUMI community structure can be analyzed considering inter-species antagonistic interactions, which are usually overlooked as you magnify the scale of the community given the inherent experimental intricacy. These studies should also be extended to test gram-negative targeting bacteriocins, as a potential strategy to control primary and opportunistic gram-negative pathogens, whose proliferation increase in various disease states (Anjana and Tiwari, 2022). Nonetheless, *in vitro* systems are usually restricted in their ability to emulate *in vivo* conditions, offering limited biological relevance. In fact, while SIHUMI forms a stable community in the intestine of mice maintained under gnotobiotic conditions (Eun et al., 2014; Buttimer et al., 2022) the relative abundances of the seven species in the murine gut after 12 weeks of colonization greatly differ from the ones obtained in LYHBHI. Hence, further exploration in more complex communities is key and should be complemented with assessment in animal models, *ex vivo* fecal fermentations, human cross-sectional and longitudinal studies and *in silico* computational tools. It is also essential to further address the challenges related to the successful establishment and sustained bacteriocin secretion by these strains within the intricate environment of the gut, before harnessing bacteriocins for targeted microbiome interventions in a precise and predictable way.

## 5 Conclusion

This study provides a comprehensive insight of the impact of bacteriocin production in a polymicrobial community context. We reveal that either broad- or narrow-spectrum bacteriocin producers exert significant effects in both targeted and non-targeted strains, considering antagonistic interactions among community members competing within the niche. While SIHUMI is a simplified consortium of human gut species, it allows to overcome the multifactorial complexity of the gut microbiome, providing reproducible results in a controlled experimental setting to unreel possible mechanisms by which bacteriocin-producing strains might shape human gut microbial communities. From a clinical perspective, the under-utilization of bacteriocins can be ascribed to a lack of awareness of their potential as precision modulators of the microbiome. Whether bacteriocins are used more extensively or not in the food and pharmaceutical industries will depend on a clear and deep understanding of how they can influence the gut microbiome in a predictable and beneficial manner.

## Supplementary Material

Supplementary file

## Figures and Tables

**Table 1:** Selective species-specific primers for SIHUMI member strains and *L. lactis* MG1363.

Target strain	Oligonucleotide Sequence (5′ to 3′)	Amplicon size	Reference
*E. faecalis* OG1RF	F: ACGGAGATTGTCACGCTTAGT R: TCGGCATTATCTGGGTGGTC	122 bp	Buttimer et al. (2022)
*E. coli* LF82	F: CGGGTGTTGTCCTAACTGCT R: CGAGTGGTCATTGGCCTCAT	107 bp	Buttimer et al. (2022)
*R. gnavus* ATCC 29149	F: GCGTGCTTGTATTCCGGATG R: GCCTGAACAGTTGCTTTCGG	115 bp	Guerin et al. (2021)
*F. prausnitzii* A2-165	F: TATTGCACAATGGGGGAAAC R: CAACAGGAGTTTACAATCCGAAG	77 bp	Lengfelder et al. (2019)
*P. vulgatus* DSM1447	F: AAGCAGCAGGGAAATGTGGA R: CTTTCCTTACTTGCGCGTCG	142 bp	Buttimer et al. (2022)
*L. plantarum* WCFS1	F: CGAAGAAGTGCATCGGAAAC R: TCACCGCTACACATGGAGTT	71 bp	Lengfelder et al. (2019)
*B. longum* ATCC15707	F: GAGGCGATGGTCTGGAAGTT R: CCACATCGCCGAGAAGATTC	108 bp	Lawley et al. (2017)
*L. lactis* MG1363	F: GCGATGAAGATTGGTGCTTGC R: ATCATCTTTGAGTGATGCAATTGC	173 bp	This work

**Table 2:** Pediocin-like bacteriocins are highlighted in red and lantibiotics in blue.

Bac+/ Bac− strains		Bacteriocin produced	Harboured plasmids	Relevant characteristics	Reference
*Lactococcus lactis* MG1363	PA1+	Pediocin PA-1	APC3490	CHL^r^, pNZ44, *pedApedD*.	Collins et al. (2018), This study
	Rum+	Ruminicin	APC3349	CHL^r^, pNZ44, *PLrumApedD*.	
	Hord+	Hordeiocin	APC2763	CHL^r^, pNZ44, *PLhrdApedD*.	
	Agil+	Agilicin	APC2764	CHL^r^, pNZ44, *PLaglApedD*.	
	20336a+	Pediocin 20336a	APC2328	CHL^r^, pNZ44, *ped20336pedD*.	
	PenA+	Penocin A	APC3488	CHL^r^, pNZ44, *PLpenApedD*.	
	Ped-	None	pNZ44	CHL^r^	
	Ltn+	Lacticin 3147	pMRCO1		Cotter et al. (2006)
	Ltn++	Lacticin 3147 (over-producer)	pMRCO1 pOM02	CHL^r^	
	Ltn−	None	pMRCO1Δ*αβ*		Cotter et al. (2003)
*Lactococcus lactis* NZ9700	Nis+	Nisin A		Nisin A-producer	de Ruyter et al. (1996), Field et al. (2008)
*Lactococcus lactis* NZ9800	Nis−	None		*L. lactis* NZ9700 Δ*nisA*	
Indicator Strains					
*Listeria innocua* DPC3572		None	pNZ44E	ERY^r^	This study
*Lactococcus lactis* MG1614		None	pNZ44E	ERY^r^	This study

CHL^r^, chloramphenicol resistance. ERY^r^, erythromycin resistance. *PL*, pediocin PA-1 leader sequence fusion gene.

## Data Availability

The original contributions presented in the study are included in the article/Supplementary material, further inquiries can be directed to the corresponding author.

## References

[R1] Anjana, Tiwari SK (2022). Bacteriocin-producing probiotic lactic acid Bacteria in controlling Dysbiosis of the gut microbiota. Front Cell Infect Microbiol.

[R2] Baquero F, Lanza VF, Baquero M-R, Del Campo R, Bravo-Vázquez DA (2019). Microcins in Enterobacteriaceae: peptide antimicrobials in the eco-active intestinal chemosphere. Front Microbiol.

[R3] Barraza DE, Ríos NS, Colombo AE, Galván LA, Minahk CJ, Bellomio A (2017). New insights into Enterocin CRL35: mechanism of action and immunity revealed by heterologous expression in *Escherichia Coli*. Mol Microbiol.

[R4] Bäuerl C, Umu ÖC, Hernandez PE, Diep DB, Pérez-Martínez G (2017). A method to assess bacteriocin effects on the gut microbiota of mice. JoVE (Journal of Visualized Experiments).

[R5] Becker N, Kunath J, Loh G, Blaut M (2011). Human intestinal microbiota: characterization of a simplified and stable Gnotobiotic rat model. Gut Microbes.

[R6] Biosystems A (2013). Creating standard curves with genomic DNA or plasmid DNA templates for use in quantitative PCR.

[R7] Bitschar K, Sauer B, Focken J, Dehmer H, Moos S, Konnerth M (2019). Lugdunin amplifies innate immune responses in the skin in synergy with host- and microbiota-derived factors. Nat Commun.

[R8] Buttimer C, Sutton T, Colom J, Murray E, Bettio PH, Smith L (2022). Impact of a phage cocktail targeting *Escherichia coli* and *Enterococcus Faecalis* as members of a gut bacterial consortium in vitro and in vivo. Front Microbiol.

[R9] Collins FWJ, Mesa-Pereira B, O’Connor PM, Rea MC, Hill C, Paul Ross R (2018). Reincarnation of bacteriocins from the *Lactobacillus pangenomic* graveyard. Front Microbiol.

[R10] Cotter PD, Draper LA, Lawton EM, McAuliffe O, Hill C, Paul Ross R (2006). Overproduction of wild-type and bioengineered derivatives of the lantibiotic lacticin 3147. Appl Environ Microbiol.

[R11] Cotter PD, Hill C, Paul Ross R (2003). A food-grade approach for functional analysis and modification of native plasmids in *Lactococcus Lactis*. Appl Environ Microbiol.

[R12] Cotter PD, Paul Ross R, Hill C (2013). Bacteriocins - a viable alternative to antibiotics?. Nat Rev Microbiol.

[R13] Davis JD, Olivença DV, Brown SP, Voit EO (2022). Methods of quantifying interactions among populations using Lotka-Volterra models. Front Syst Biol.

[R14] de Ruyter PG, Kuipers OP, de Vos WM (1996). Controlled gene expression Systems for *Lactococcus Lactis* with the food-grade inducer nisin. Appl Environ Microbiol.

[R15] Dedrick S, Warrier V, Lemon KP, Momeni B (2023). When does a Lotka-Volterra model represent microbial interactions? Insights from in vitro Nasal Bacterial Communities. mSystems.

[R16] Dicks LMT, Dreyer L, Smith C, van Staden AD (2018). A review: the fate of bacteriocins in the human gastro-intestinal tract: do they cross the gut-blood barrier?. Front Microbiol.

[R17] Draper LA, Grainger K, Deegan LH, Cotter PD, Hill C, Paul Ross R (2009). Cross-immunity and immune mimicry as mechanisms of resistance to the Lantibiotic Lacticin 3147. Mol Microbiol.

[R18] Eun CS, Mishima Y, Wohlgemuth S, Liu B, Bower M, Carroll IM (2014). Induction of bacterial antigen-specific colitis by a simplified human microbiota consortium in gnotobiotic interleukin-10^-/-^ mice. Infect Immun.

[R19] Faust K, Raes J (2012). Microbial interactions: from networks to models. Nat Rev Microbiol.

[R20] Field D, Begley M, O’Connor PM, Daly KM, Hugenholtz F, Cotter PD (2012). Bioengineered nisin A derivatives with enhanced activity against both gram positive and gram negative pathogens. PLoS One.

[R21] Field D, Connor PMO, Cotter PD, Hill C, Paul Ross R (2008). The generation of nisin variants with enhanced activity against specific gram-positive pathogens. Mol Microbiol.

[R22] Field D, Fernandez M, de Ullivarri R, Ross P, Hill C (2023). After a century of nisin research - where are we now?. FEMS Microbiol Rev.

[R23] Gilbert JA, Blaser MJ, Gregory Caporaso J, Jansson JK, Lynch SV, Knight R (2018). Current understanding of the human microbiome. Nat Med.

[R24] Guerin E, Shkoporov AN, Stockdale SR, Comas JC, Khokhlova EV, Clooney AG (2021). Isolation and characterisation of ΦcrAss002, a crAss-like phage from the human gut that infects *Bacteroides Xylanisolvens*. Microbiome.

[R25] Guinane CM, Cotter PD (2013). Role of the gut microbiota in health and chronic gastrointestinal disease: understanding a hidden metabolic organ. Ther Adv Gastroenterol.

[R26] Guinane CM, Lawton EM, O’Connor PM, O’Sullivan Ó, Colin Hill R, Ross P (2016). The bacteriocin bactofencin A subtly modulates gut microbial populations. Anaerobe.

[R27] Heilbronner S, Krismer B, Brötz-Oesterhelt H, Peschel A (2021). The microbiome-shaping roles of bacteriocins. Nat Rev Microbiol.

[R28] Holo H, Nes IF (1989). High-frequency transformation, by electroporation, of Lactococcus Lactis Subsp Cremoris grown with Glycine in osmotically stabilized media. Appl Environ Microbiol.

[R29] Iancu C, Grainger A, Field D, Cotter PD, Hill C, Paul Ross R (2012). Comparison of the potency of the lipid II targeting antimicrobials nisin, lacticin 3147 and vancomycin against gram-positive Bacteria. Probiotics Antimicrob Prot.

[R30] Johnson EM, Jung DY-G, Jin DY-Y, Jayabalan DR, Yang DSH, Suh JW (2018). Bacteriocins as food preservatives: challenges and emerging horizons. Crit Rev Food Sci Nutr.

[R31] Koc F, Sugrue I, Murphy K, Renzetti S, Martijn Noort R, Ross P (2022). The microbiome modulating potential of superheated steam (SHS) treatment of dietary Fibres. Innovative Food Sci Emerg Technol.

[R32] Kommineni S, Bretl DJ, Lam V, Chakraborty R, Hayward M, Simpson P (2015). Bacteriocin production augments niche competition by enterococci in the mammalian gastrointestinal tract. Nature.

[R33] Krause JL, Schaepe SS, Fritz-Wallace K, Engelmann B, Rolle-Kampczyk U, Kleinsteuber S (2020). Following the community development of SIHUMIx - a new intestinal in vitro model for bioreactor use. Gut Microbes.

[R34] Lawley B, Munro K, Hughes A, Hodgkinson AJ, Prosser CG, Lowry D (2017). Differentiation of Bifidobacterium Longum subspecies Longum and Infantis by quantitative PCR using functional gene targets. PeerJ.

[R35] Lengfelder I, Sava IG, Hansen JJ, Kleigrewe K, Herzog J, Neuhaus K (2019). Complex bacterial consortia reprogram the colitogenic activity of Enterococcus Faecalis in a Gnotobiotic mouse model of chronic, immune-mediated colitis. Front Immunol.

[R36] Medeiros-Silva J, Jekhmane S, Paioni AL, Gawarecka K, Baldus M, Swiezewska E (2018). High-resolution NMR studies of antibiotics in cellular membranes. Nat Commun.

[R37] Murphy EF, Cotter PD, Hogan A, O’Sullivan O, Joyce A, Fouhy F (2013). Divergent metabolic outcomes arising from targeted manipulation of the gut microbiota in diet-induced obesity. Gut.

[R38] Navarro SA, Lanza L, Rios Colombo NS, Fernandez de Ullivarri M, Acuña L, Sosa-Padilla B (2019). Obtaining an Ent35-MccV derivative with mutated hinge region that exhibits increased activity against Listeria monocytogenes and Escherichia Coli. Appl Microbiol Biotechnol.

[R39] O’Callaghan A, van Sinderen D (2016). Bifidobacteria and their role as members of the human gut microbiota. Front Microbiol.

[R40] O’Connor PM, Kuniyoshi TM, Oliveira RPS, Hill C, Ross RP, Cotter PD (2020). ‘Antimicrobials for food and feed; a Bacteriocin perspective’. Current opinion in biotechnology, plant biotechnology ●. Food Biotechnol.

[R41] O’Reilly C, Grimaud GM, Coakley M, O’Connor PM, Mathur H, Peterson VL (2023). Modulation of the gut microbiome with Nisin. Sci Rep.

[R42] Qin J, Li R, Raes J, Arumugam M, Burgdorf KS, Manichanh C (2010). A human gut microbial gene catalogue established by metagenomic sequencing. Nature.

[R43] Relman DA (2012). The human microbiome: ecosystem resilience and health. Nutr Rev.

[R44] Ríos Colombo MC, Chalón FG, Dupuy CF, Gonzalez, Bellomio A (2019). The case for class II Bacteriocins: A biophysical approach using “suicide probes” in receptor-free hosts to study their mechanism of action. Biochimie.

[R45] Ríos Colombo NS, Chalón MC, Navarro SA, Bellomio A (2017). Pediocin-like Bacteriocins: new perspectives on mechanism of action and immunity. Curr Genet.

[R46] Rooks MG, Garrett WS (2016). Gut microbiota, metabolites and host immunity. Nat Rev Immunol.

[R47] Shah BR, Li B, Al Sabbah H, Wei X, Mráz J (2020). Effects of prebiotic dietary fibers and probiotics on human health: with special focus on recent advancement in their encapsulated formulations. Trends Food Sci Technol.

[R48] Silva CCG, Silva SPM, Ribeiro SC (2018). Application of bacteriocins and protective cultures in dairy food preservation. Front Microbiol.

[R49] Sokol H, Pigneur B, Watterlot L, Lakhdari O, Bermúdez-Humarán LG, Gratadoux J-J (2008). Faecalibacterium Prausnitzii is an anti-inflammatory commensal bacterium identified by gut microbiota analysis of Crohn disease patients. Proc Natl Acad Sci.

[R50] Taylor M (2014). Handbook of natural antimicrobials for food safety and quality.

[R51] Tropini C, Earle KA, Huang KC, Sonnenburg JL (2017). The gut microbiome: connecting spatial organization to function. Cell Host Microbe.

[R52] Venturelli OS, Carr AV, Fisher G, Hsu RH, Lau R, Bowen BP (2018). Deciphering microbial interactions in synthetic human gut microbiome communities. Mol Syst Biol.

[R53] Weiss AS, Niedermeier LS, Von Strempel A, Burrichter AG, Ring D, Meng C (2023). Nutritional and host environments determine community ecology and keystone species in a synthetic gut bacterial community. Nat Commun.

