# Supplementary material for: Impact of bacteriocin-producing strains on bacterial community composition in a simplified human intestinal microbiota
Source: Front Microbiol. Author manuscript; Available in PMC 2024 Jan 11. (PMC10748383; doi:10.3389/fmicb.2023.1290697)
Supplement: Supplementary file [file EMS192682-supplement-Supplementary_file.pdf]

Supplementary Material

Supplementary Tables

Supplementary Table 1. Genome size (bp) of SIHUMI member strains and *L. lactis* MG1363. The mass of each genome was calculated multiplying the genome size by 1.096×10^{-12} (average Molecular Weight of 1 bp in ng).

Strain	Genome size (bp)	Genome Mass (ng)
***E. faecalis* OG1RF**	2739625	3.00×10^{-6}
***E. coli* LF82**	4773108	3.00×10^{-6}
***R. gnavus* ATCC 29149**	3549191	3.89×10^{-6}
***F. prausnitzii* A2-165**	3102523	3.40×10^{-6}
***P. vulgatus* DSM1447**	4773108	5.23×10^{-6}
***B. longum* ATCC15707**	2385164	2.61×10^{-6}
***L. plantarum* WCFS1**	3308274	3.63×10^{-6}
***L. lactis* MG1363**	2529478	2.77×10^{-6}

Supplementary Figures

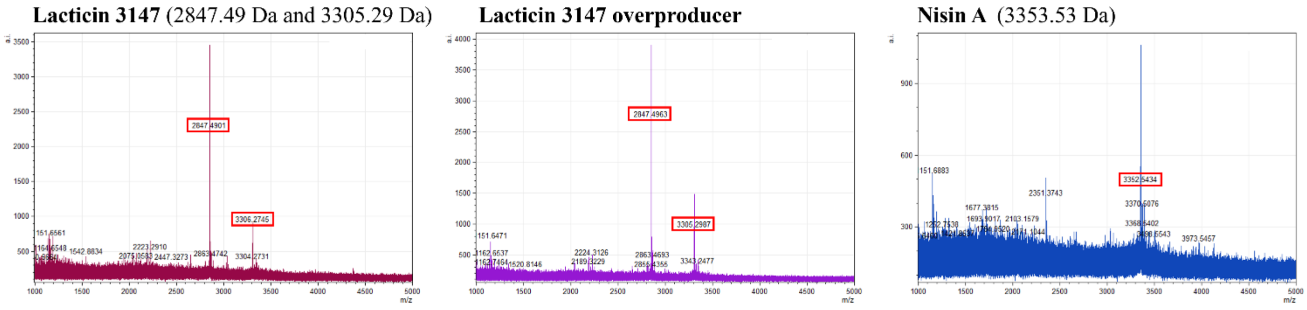

Supplementary Figure 1. Colony Mass Spectrometry results of the lantibiotic-producing strains. Each graph indicates the bacteriocin and the predicted mass in brackets. The measured mass in Daltons is highlighted in red. The peaks corresponding to each bacteriocin were absent in the isogenic non-producing (Bac-) strains (data not shown).

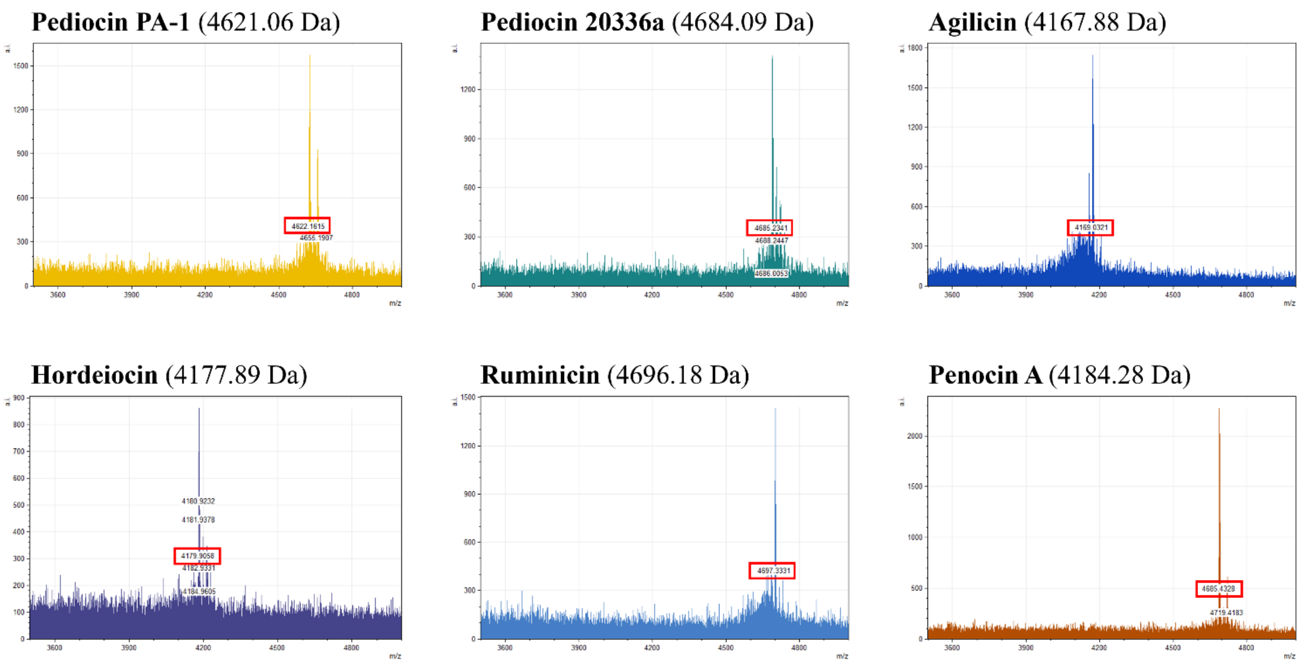

Supplementary Figure 2. Mass Spectrometry results of cell-free supernatants of the pediocin-producing strains after a two-step purification procedure. Each graph indicates the bacteriocin and the predicted mass in brackets. Predicted masses were adjusted for the presence of disulphide bonds. The measured mass in Daltons is highlighted in red. The peaks corresponding to each bacteriocin were absent in the isogenic non-producing (Bac-) strain (data not shown).

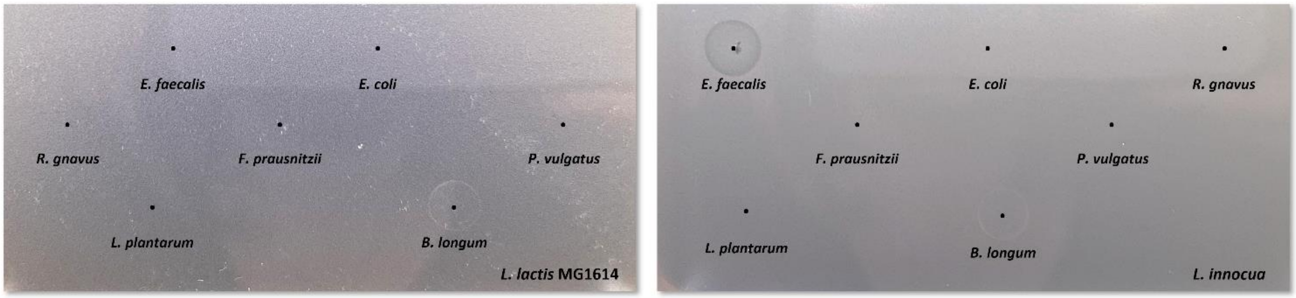

Supplementary Figure 3. Antimicrobial activity of culture supernatants from SIHUMI strains by agar spot diffusion method against the indicator organism *L. lactis* MG1614 (left) and *L. innocua* (Right). The black dots indicate where each supernatant was spotted.

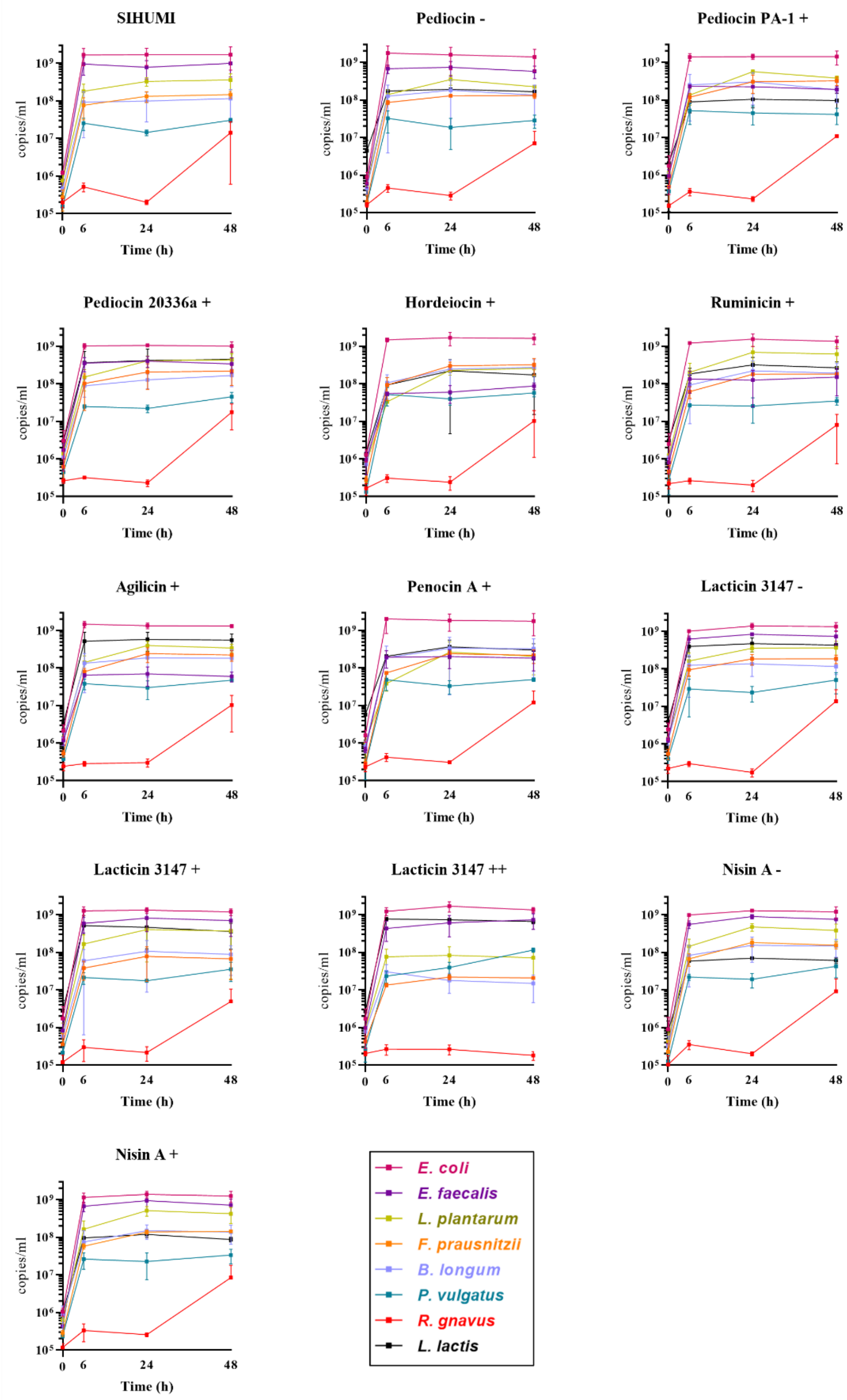

Supplementary Figure 4. Genome copies/ml over time (0, 6, 24 and 48 h) of members of SIHUMI consortium in LYHBHI after inoculation with Bac+ and Bac- *L. lactis* strains at time 0. Each graph indicates the bacteriocin produced. Each time point is represented as a mean with standard deviation of 4 replicates.

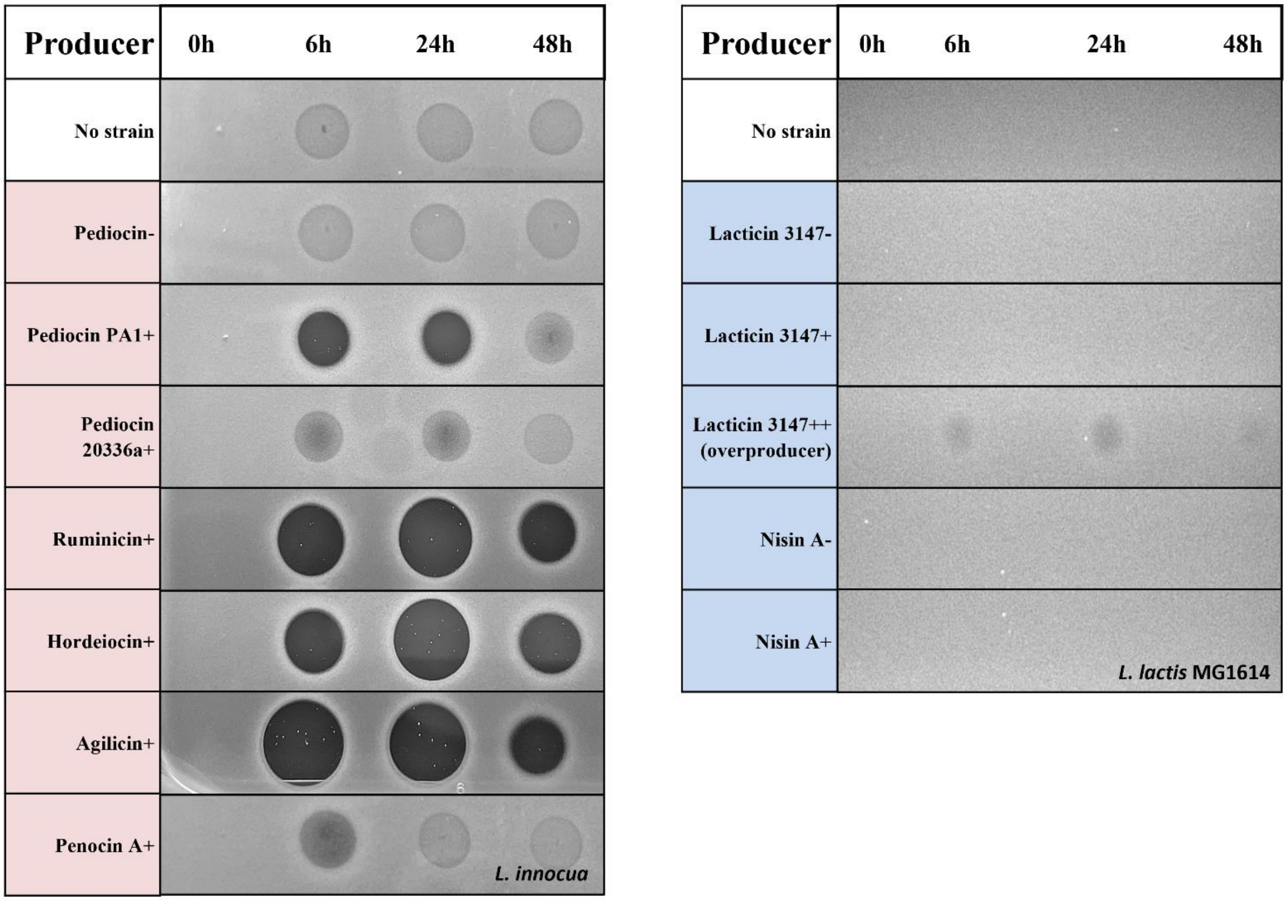

Supplementary Figure 5. Spot assays of SIHUMI culture supernatants at different time points (0, 6, 24 and 48 h) against *L. innocua* (for pediocin-like bacteriocins) and *L. lactis* MG1614 (for lantibiotics).
